# Evaluating the integration of pre-mortem body donor imaging into a dissection-based medical anatomy course

**DOI:** 10.1186/s12909-020-02300-4

**Published:** 2020-10-23

**Authors:** Kimberly McBain, Brandon Azimov, Jeremy O’Brien, Geoffroy P. J. C. Noël, Nicole M. Ventura

**Affiliations:** 1 School of Physical and Occupational Therapy, McGill University, Montreal, Quebec Canada; 2 Department of Diagnostic Radiology, McGill University, Montreal, Quebec Canada; 3 Division of Anatomical Sciences, Department of Anatomy and Cell Biology, McGill University, Strathcona Anatomy and Dentistry Building, 3640 University Street, Montreal, QC H3A 0C7 Canada; 4 Institute of Health Sciences Education, Faculty of Medicine, McGill University, Montreal, Quebec Canada

**Keywords:** Anatomy education, Anatomy, radiology, Pre-mortem, Diagnostic imaging, Anatomical dissection

## Abstract

**Background:**

Medical faculties are currently embracing a modernistic approach to anatomical education that integrates diagnostic imaging largely through post-mortem computed tomography scanning of body donors. Post-mortem imaging, however, poses a multitude of challenges. The purpose of this study was to assess the implementation of pre-mortem donor-specific diagnostic imaging on student learning and dissection experience in addition to understanding the potential impact on students’ preparation for clinical practice.

**Methods:**

Students in a fourth-year medicine elective course were divided into groups; group 1 received pre-mortem donor-specific diagnostic imaging, while group 2 received pathology-specific diagnostic imaging, a collection of images relating to the type(s) of pathologies the donors exhibited, though not specific to the donors themselves. Both groups also received a donor-specific case vignette. A convergent, parallel mixed methods design was employed. This included integrating data from group responses to a study participant survey and students’ academic assessment scores analyzed quantitatively through statistical analyses with data from focus group sessions investigating the psychosocial aspects of the student dissection experience and perceptions of the imaging use in the course analyzed qualitatively.

**Results:**

As compared to students receiving pathology-specific diagnostic imaging, the quantitative results demonstrated that students receiving pre-mortem donor-specific diagnostic imaging more positively supported the relevancy of diagnostic imaging to their understanding of anatomy, valued the integration for future practice, and suggested an earlier integration within their medical curriculum. Qualitatively, two main themes were observed: the influence of diagnostic imaging integration on dissection experience and on professional mindset. Although both student groups received imaging corresponding to their body donor, consideration towards the humanistic nature of the body donor as a patient with a history was limited to student feedback from the donor- specific diagnostic imaging group.

**Conclusion:**

Overall the integration of pre-mortem donor-specific diagnostic imaging into anatomical dissection provided students with practical skill development, an enhanced dissection experience, and reinforced personal qualities critical for future practice.

## Background

Given the advancements in diagnostic imaging and its extensive use across multiple medical specialties, the integration of diagnostic imaging and anatomy together has become an imperative component of medical school curricula globally [1–4]. In fact, early exposure to diagnostic imaging has been shown to provide medical students with the ability to interpret basic radiographs and improve their understanding of three-dimensional human anatomy [5]. In efforts to increase the exposure of medical students to radiology, multiple studies have focused on effective ways to integrate diagnostic imaging into anatomical instruction, more specifically into dissection-based anatomy courses [1, 3, 6, 7]. Anatomy taught with radiological images has been shown to enhance the anatomical learning experience by allowing the students to comprehend the material more efficiently [5], leading to positive shifts in the students’ academic performance [5, 8, 9]. With this in mind, many medical faculties are embracing a modernistic approach to the teaching of anatomy that integrates radiology into the anatomy laboratory largely through post-mortem computed tomography (CT) scanning of body donors presenting with a variety of pathologies [10–12]. Though this pedagogical tactic has been proven to provide students with valuable learning opportunities [10, 12], there are still many challenges to this approach. These challenges include post-mortem state and scanning artifacts [7], the timeframe in which body donors need to be scanned prior to embalming [7, 13–15], the expense of scanning [10], and limitations to the imaging techniques used for post-mortem scanning [15]. Specifically, some studies have observed unwanted changes to the head, neck, and torso of CT scans of cadavers due to the embalming process and images being taken post-mortem [7]. For formalin-embalmed cadavers, the deterioration of image quality after embalming may be a result of the fixation of brain tissue caused by high concentrations of formaldehyde [16]. When comparing imaging quality pre- versus post-embalming using a variety of embalming fluids such as Thiel, formalin, Genelyn, and Imperial College London- Soft Preserving solutions, with various types of imaging modalities including plain radiography, ultrasound and CT of human cadavers, it has been suggested that while there is no ideal embalming fluid, formalin has shown to offer the least benefit to improve imaging quality particularly after embalming [16]. Given these challenges, alternative strategies to incorporate radiology into medical curricula should be evaluated using appropriate scientific methods.

There are two main formats of radiological imaging which have been used to complement anatomy teaching; pathology-specific diagnostic imaging (PDSI), and donor-specific diagnostic imaging (DSDI). Generic or PSDI refers to the collection of imaging that is often acquired by faculty to complement their teaching in anatomy, demonstrating normal anatomy and common regional pathologies [17]. Generally, PSDI is of diagnostic quality and can be sourced from past patient cases or from available internet sources. It is therefore, not specific to body donors students would be studying from in the anatomy laboratory. With PSDI being readily available, it does allow faculty to assimilate relatable imaging that can be re-used on an annual basis, eliminating the labour associated with acquiring and interpreting body donor imaging every time there is a turn-over of bodies being used. In contrast, DSDI describes radiological imaging that is exclusive to individual cadaveric body donors and as mentioned above, is often acquired post-mortem [10–12]. This imaging intervention is advantageous in that it provides students with the opportunity to review radiological imaging that may illustrate anatomical variations or a mixture of pathologies that can later be observed during the students’ dissections. While the anatomical variations or pathologies students may be exposed to is highly dependent on what the body donors present with, many common pathologies typically re-occur yearly, allowing for some consistency in the annual collection of DSDI [18]. While integrating post-mortem DSDI may appear to be the most feasible, the optimization of high-quality radiological imaging should be prioritized. The incorporation of pre-mortem DSDI, however, would further allow students to work with radiological images of diagnostic quality removing the aforementioned complications associated with post-mortem imaging. Pre-mortem DSDI may better facilitate the translation of future clinical practice as medical students would have the opportunity to practice interpreting the type of radiological imaging they would be exposed to in the clinical setting. The evaluation however of pre-mortem DSDI into anatomical dissection for medical students has yet to be investigated.

Unique to the province of Québec is the Dossier Santé Québec (DSQ), a tool which serves as a patient history cataloguing system in which patient imaging across Québec health institutions is collected. Driven by the need to address the abovementioned challenges associated with post-mortem DSDI, pre-mortem DSDI was collected from the DSQ for this investigation. This investigation therefore aimed to assess the implementation of diagnostic quality, pre-mortem donor-specific imaging into a cadaveric-dissection-based course at McGill University entitled Anatomy for Surgeons (AFS). Through the comparison of student interaction with pre-mortem DSDI against student interactions with diagnostic imaging not specific to a body donor, the authors sough to determine whether pre-mortem, DSDI is a useful tool that would enhance the students’ perceived improvement in anatomy knowledge and/or their dissection experience as well as positively influence their preparation and medical training for their future careers.

## Methods

### Course design and prior anatomy and radiology knowledge

The AFS course at McGill University, Montreal, Canada, is a 4-week, elective course offered to 35 fourth-year medical students. It is designed for students embarking on careers in surgery, pathology or radiology to acquire a detailed hands-on exposure to human anatomy, further building on the content they learned during their pre-clinical years. Components of the AFS course include detailed, regional, cadaveric dissections, literature reviews on anatomical variations, imaging seminars and participation in conference presentations given by clinical experts in a variety of surgical specialties. On average, students spend 16.5 h a week dissecting human cadavers in groups of 5 students per one body donor, a total of 66 h over the 4-week period. The breakdown of dissection groups is described in detail below. All regional dissections closely followed the approaches of *Gray’s Clinical Photographic Dissector of the Human Body*, with some modifications to allow students to attempt and practice surgical approaches they may encounter in their respective surgical residency field. Some of these procedures included cricothyrotomy, lateral canthotomy, thoracostomy, posterior approach of kidney, knee arthrocentesis, anterior approach of the hip joint and carpal tunnel release. Anatomy faculty members acted as facilitators to guide student groups through their dissections.

Aside from the AFS course, McGill medical students are also exposed to anatomy and radiology topics in the Fundamentals of Medicine and Dentistry (FMD) course offered during the first and second years of their undergraduate medical education curriculum. Students learn anatomy through a systems-based approach, participating in 31 h of lecture and 55 h of cadaveric-dissection based anatomy laboratories (with few laboratories being supplemented with prosections). During each of the systemic blocks of FMD, a team of radiologists provide a 10–15 min radiology presentation in the anatomy laboratory, covering both normal anatomy and common pathologies associated with the related system. Further exposure to radiology occurs during clinical rotations during the students’ clerkship year.

### Study population

The study population for this investigation consisted of students registered for AFS in the winter 2018 academic semester. Students were pre-assigned to one of 7 dissection groups (5 students per dissection group, total *n* = 35) and a regional stream based on their surgical residency program of interest. These interests were indicated on the students’ applications when registering for the AFS elective. The regional streams consisted of head and neck (1 student per dissection group, *n* = 7), trunk (2 students per dissection group, *n* = 14), and limb (2 students per dissection group, *n* = 14) streams. Each dissection group was allocated one body donor and all students within the group actively participated in the weekly scheduled dissections. All dissection groups received a clinical case vignette created by a faculty member of the Department of Diagnostic Radiology at McGill University (J.O.) based on the detailed donor-specific medical and imaging history acquired from the DSQ and the set of coinciding diagnostic images, such as CT, CT-angio, chest X-ray or MRI scans obtained at varying periods throughout the life of the body donor. For this investigation, dissection groups were further divided based on the type of imaging intervention received and timing of the receipt of the diagnostic imaging in the AFS course. Of the 7 dissection groups, 4 groups received pre-mortem radiological images specific to their assigned body donor as well as their case vignette at the beginning of the AFS course; hereby to be labelled as the donor-specific diagnostic imaging (DSDI) dissection group (*n* = 20). The latter 3 dissection groups received a collection of diagnostic images that coincided with the donor-specific case received yet the images themselves were not donor specific; hereby referred to as the pathology-specific diagnostic imaging (PSDI) dissection group (*n* = 15). Additionally, this group of students received both the clinical case scenario and their collection of diagnostic imaging at the beginning of the third week of the four-week AFS course. The difference in the timing of receipt of the images ensured that the PSDI dissection groups did not make significant mistakes in the approach to their dissection given that the diagnostic images provided were not images specific to their body donor. While the assignment to the regional dissection streams was by student choice based on interest, the assignment to DSDI or PSDI dissection groups was done at random. Additionally, students had the chance to work in their small dissection groups alongside a radiology resident (post-graduate years 3–4) to discuss their findings and ask questions to allow for assistance with imaging interpretation. This question and answer period was held 1-week after student groups received their imaging to give them time to view the images and interpret them independently. As a component of the AFS course requirements, students were asked to compare their donor-specific clinical case vignette, collection of PSDI or DSDI, and their cadaveric dissection to be able to present a list of donor-specific pathologies and a differential diagnosis for their body donor. The diagnostic imaging collections for all DSDI and PSDI dissection groups were uploaded to iPads in the anatomy laboratory for easy student access. Lastly, those students willing to voluntarily participate in this investigation (study participants) were asked to also complete a consent form. The number of study participants and the extent of their involvement in the study are described below.

### Research design

A convergent parallel mixed methods design was employed using both quantitative and qualitative data sets (see Table 1) with a pragmatist epistemological stance. Pragmatist discourse has been commonly cited in mixed methods literature as a philosophical approach that considers more than a single scientific method [19]. In this investigation, the objective was to receive student feedback in the form of responses to a study participant survey, analyzed quantitatively and in the form of student focus group sessions, analyzed qualitatively. Pragmatic measures, as those used in this study, prioritize the perspective of the user in the data collected and favour measures that assess the application of the intervention to practice [20]. Key features of pragmatic studies that this investigation honed in on were to examine questions that were important for stakeholders making decisions regarding radiological imaging use in medical curricula, to evaluate the implications of integrating pre-mortem DSDI in an anatomy laboratory setting, modelling its use in practice, and to examine the integration of this imaging intervention in comparison to traditional generic, PSDI [20].

**Table 1:** Representation of the Pragmatic Epistemological Stance and Mixed Methods Study Design

	Quantitative Data Sets	Qualitative Data Sets
**Methods of Collection:**	• Study participant survey^a^	• Post-course focus group sessions
	• Assessment of student academic scores.	• Transcribed audio-recordings of focus group sessions
**Methods of Analysis:**	• Mann-Whitney U Test	• Inductive and deductive coding with thematic triangulation using ATLAS.ti Software
	• Descriptive statistics: mean, median, standard deviation	
	• Two-tailed Student’s *t*-test	
	*All computed using GraphPad Prism Software*	

^a^Survey adapted from Tumerzi et al. [21]. Clinical Anatomy and Bohl et al. [13]. Clinical Anatomy

The quantitative methods used for this investigation were well suited to measure differences in the students’ experience, correlating their respective radiological imaging with their cadaveric dissection. Additionally, student performance on anatomy-specific assessments was assessed quantitatively. The qualitative method, a semi-structured focus group discussion, was best suited to gain an in-depth understanding of the students’ perspectives and experiences in using the two different imaging interventions to complement their anatomical dissection. The focus group discussion was chosen for this investigation, whereby the researcher (NMV) adopted the role of a facilitator, generating discussion amongst the group and intervening with questions and prompts when necessary in order to facilitate said discussion [22]. This method is different from a group interview where the researcher has a more dominant role as an interviewer generating questions directed to specific individuals or to the group as a whole [22, 23]. The objective of the focus group session was to provoke discussion amongst a group of people with a shared experience to consider their thoughts, perspectives and opinions within a casual conversational format [23]. In this format, participants were welcome to contribute to the facilitated discussions and provide their own insight comfortably. Overall, this mixed methods study design provided an enhanced understanding of the phenomenon surrounding the research objectives for this investigation. Quantitative and qualitative data sets were integrated at the analysis and interpretation stages of the study.

### Data collection

Of the 20 students assigned to the DSDI dissection group and the 15 students assigned to the PSDI dissection group, 15 (75%) and 11 (73%) students consented to participate in the study respectively. The demographic information of the study participants willing to disclose is as follows: the DSDI group comprised of 9 females and 6 males with an average age of 26.17 ± 1.90, the PSDI group comprised of 3 females and 7 males (one student chose not to disclose) with an average age of 26.13 ± 2.53.

#### Quantitative data collection

All study participants (total *n* = 26; DSDI *n* = 15; PSDI *n* = 11) were asked to complete a study participant survey adapted from those of Turmezei and colleagues [21] and Bohl and colleagues [13] at the end of the AFS course. This survey was designed to quantitatively measure aspects of the student experience and their satisfaction surrounding the use of diagnostic imaging in cadaveric dissection. Additionally, practical examination and dissection related course grades of all study participants were considered in order to measure any potential differences in academic performance between groups. These assessments included evaluation of the quality of the students’ dissections, an oral laboratory presentation, whereby students presented the anatomy of a specific region of their dissection to a faculty member, and an oral laboratory exam where anatomy faculty members questioned students on the anatomy of the regions of the dissection that the student did not present. These questions ranged from identification, structural and functional anatomy questions, to probing students on the anatomy relating to various surgical approaches and anatomical relationships. Questions relating to radiology were not included in these assessments.

#### Qualitative data collection

Both imaging groups separately participated in a 40–45 min semi-structured focus group session at the end of the AFS course (*n* = 26; DSDI *n* = 15; PSDI *n* = 11). These discussions were done to gain a profound understanding of the psychosocial aspects of the students’ dissection experience within each group as well as to obtain an indication of how the students perceived the use of this tool may influence their medical training and preparation for future clinical practice. Both focus group sessions were audio recorded. Consent to record was verbally obtained by study participants during the sessions. Both audio recorded sessions were professionally transcribed.

### Data analysis

#### Quantitative data analysis

Statistical analyses were performed using Prism 7.0a software (GraphPad Software Inc., La Jolla, CA, USA). Data collected from the study participant survey was analyzed using non-parametric statistics; an unpaired, Mann Whitney U test. Descriptive statistics including mean (M), median (Md), and standard deviation (SD) were computed for all survey items. In order to measure internal consistency and reliability of survey items, a Cronbach’s alpha value was calculated using IBM SPSS Statistics for Macintosh, version 26.0 software (IBM Corp, Armonk, NY, USA). After the removal of the two survey items ‘*I had appropriate assistance in the [question and answer period] with interpreting radiology images*’ and ‘*Overall, I feel that radiology is covered in enough detail throughout the medical curriculum,*’ a Cronbach’s alpha value of 0.703 was achieved. This Cronbach’s alpha value is within the accepted range, indicating internal consistency between survey items. These two removed survey items were intended to provide the authors with course and curricular feedback, but were unrelated to the research objectives [24]. Lastly, student academic assessment scores were parametrically compared by unpaired, two-tailed Student’s *t*-test and the data presented as mean ± SD. For all of the statistical analyses, a *P* ≤ 0.05 was deemed statistically significant.

#### Qualitative data analysis

The analysis of qualitative data for this investigation was conducted using ATLAS.ti software, version 8.2.4 (Scientific Software Development GmbH, Berlin, Germany). Thematic analysis was used to identify, analyze, and report themes within the recorded transcripts for each of the focus group sessions [25]. Thematic coding was completed for qualitative description using constant comparative methods borrowed from grounded theory [25]. Two independent coders (B.A and K.M) reviewed the transcripts inductively to generate codes. Codes were continuously revised, and consensus was achieved between the two independent coders. Once consensus was achieved, three independent reviewers (B.A, K.M and NMV) grouped emergent codes into overarching themes representing all of the qualitative data collected [26]. Triangulation of evidence from different individuals helps to increase the credibility of key themes elicited from the qualitative data [26]. Themes can be defined as patterned responses or meanings from the text that are deemed as important and relevant to our research objectives [27]. Furthermore, the process of inductive coding was used to allow codes to emerge from the data alone followed by deductive coding. The deductive coding method allowed for identification of the data relating to our research questions and the items listed in the study participant survey.

### Ethical considerations

In order to access patient donor imaging for the purposes of anatomical teaching in the anatomy laboratory, legal consent was obtained to access relevant pre-mortem imaging from the DSQ by both the Québec Ministry of Health and by the body donors and/or their families. All pre-mortem body donor images collected were also anonymized by a committee comprising of anatomy and radiology faculty members at McGill University who oversee the use of all body donor imaging for educational use to protect patient privacy. Furthermore, students in the AFS course were able to access all anonymized imaging collections regardless of their dissection group assignment for personal study and to ensure equity of their experiences. All study participants voluntarily consented to partake in this investigation via a written consent form at the beginning of the AFS course. Follow-up consent was also acquired verbally during focus group sessions. Lastly, all data was analyzed following completion of the course and students were ensured that their choice to take part in this investigation, or not, would have no consequence on their academic success in the AFS course.

## Results

### Quantitative results

#### DSDI and PSDI dissection groups were equally satisfied with various aspects of imaging integration within the course

As shown in Table 2, the following survey items (items 1–3) demonstrated no statistical significant differences; ‘*the radiology images provided were clear and easy to interpret’*, *‘I would like to see more donor images with pathological findings’*, and *‘I feel more confident in my ability to recognize anatomical structures after correlating my dissection with radiology images provided’* (all *P* > 0.05). These results demonstrate that both the DSDI and PSDI dissection groups agreed upon the quality and clarity of the radiology images, strongly agreed to an appreciation of increased exposure to donor imaging with pathological findings and, despite the differences in imaging provided to both groups, shared a perceived increase in confidence in identifying anatomical structures on radiological images following the dissection experience. Although survey item 4, *‘the radiology images provided were relevant to the dissection I completed’* also showed insignificant results (*P* > 0.05), DSDI group students were more likely to agree with this statement whereas PSDI group students were more likely to respond neutrally to this item. Furthermore, students in both groups either responded with ‘strongly agree’ or ‘agree’ to survey item 5, ‘*this was an appropriate time within the medical curriculum to integrate radiology into anatomical dissection’* (*P* > 0.05).

**Table 2:** Median (Md), Mean (M), and Standard-Deviations (SD) of Student Participant Survey Scores

	Donor-Specific Diagnostic Imaging Group			Pathology-Specific Diagnostic Imaging Group			Mann-Whitney U	***P***
Subject Survey Questions								
	Md	M	SD	Md	M	SD		
1. The radiology images provided were clear and easy to interpret.	4.00	4.33	0.49	4.00	4.18	0.60	72.50	0.71
2. I would like to see more donor images with pathological findings	5.00	4.93	0.26	5.00	4.82	0.40	73.00	0.56
3. I feel more confident in my ability to recognize anatomical structures after correlating my dissection with radiology images provided	4.00	3.60	0.99	3.50	3.40	0.97	67.00	0.66
4. The radiology images provided were relevant to the dissection I completed.	4.00	3.50	1.23	3.00	3.00	1.41	60.50	0.37
5. This was an appropriate time within the medical curriculum to integrate radiology into anatomical dissection.	4.00	4.00	1.00	4.00	3.55	1.04	62.00	0.30
6. The radiology images complemented my understanding of relevant anatomy.	4.00	4.07	1.03	3.00	3.27	0.79	42.00	0.03*
7. I would like to see cadaver-based radiology integrated earlier in the medical curriculum.	5.00	4.87	0.52	5.00	4.36	0.92	52.00	0.05*
8. The exposure to digital imaging will help me in my future clinical practice.	4.00	4.29	0.73	5.00	4.36	0.81	71.00	0.74
9. Overall I feel that integrating radiology with anatomical dissection is relevant to my future practice and valuable for student learning.	5.00	4.87	0.35	4.00	4.36	0.50	41.00	0.01*
10. Integrating radiology into the dissection made my donor feel like a patient.	5.00	4.40	0.74	4.00	4.00	0.77	58.50	0.20

‘*’ indicates statistical significance (*P* ≤ 0.05). Likert Scale: Strongly Agree (5), Agree (4), Neutral (3), Disagree (2), Strongly Disagree (1)

#### Students with DSDI agree that imaging was complementary to their understanding of anatomy and strongly agreed for earlier integration in program

While both groups of students were equally as satisfied with the general aspects of diagnostic imaging in the AFS course, statistically significant differences were observed when assessing survey items related to the implementation of these images into practice; items 6 (*P* < 0.05) and 7 (*P* = 0.05). Student responses to survey item 6 regarding the complementarity of images to the relevant anatomy revealed that the DSDI dissection groups felt as though their imaging more closely related to their dissection and understanding of anatomy as compared to students in the PSDI dissection group. Furthermore, responses to survey item 7 regarding earlier curricular integration of DSDI demonstrated that DSDI dissection groups were more inclined to agree that cadaver-based radiology could be implemented into earlier years of the undergraduate medical curriculum; PSDI dissection groups were less inclined to agree with this statement. As previously mentioned, however, both groups of students suggested that this was the appropriate time in the medical curriculum to integrate radiology into the anatomy laboratory.

#### DSDI dissection groups recognize the application for future clinical practice

The comparison of DSDI and PSDI dissection group responses to survey item 8, *‘the exposure to digital imaging will help me in my future clinical practice’* (*P* > 0.05) revealed insignificant results as both student groups agreed to the relevancy of this exercise towards their future careers as physicians. Contrary to this, a statistically significant difference was observed for survey item 9, *‘overall, I feel that integrating radiology with anatomical dissection is relevant to my future practice and is valuable for student learning’* (*P* < 0.05). The differences between group responses however related to a consensus; the DSDI dissection groups responding with ‘strongly agree’ while the majority of PSDI dissection groups responded with ‘agree’. Additionally, survey item 10, ‘*integrating radiology into the dissection component made my donor feel more like a patient’* (*P* > 0.05) demonstrated mixed results. While a consensus of ‘strongly agree’ was observed in the DSDI dissection group, there were varied responses from the PSDI dissection groups ranging between ‘agree’ and ‘strongly agree.’ As demonstrated by these survey items, DSDI dissection groups had a greater understanding of the relevancy of this exercise to their future clinical practice and were more likely to respond ‘strongly agree’ to the survey item addressing their view of the body donor as a “patient.”

#### Academic assessment scores were not influenced by the type and timing of imaging provided

**Fig. 1:**
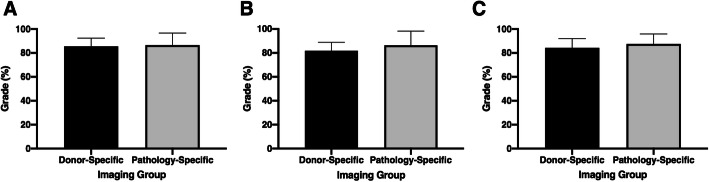
Data represents the student academic assessment scores for dissection quality (**a**), oral examination (**b**) and oral presentation (**c**)

The outcome of the statistical comparison for the following assessment scores was insignificant (*P* > 0.05); the oral laboratory exam (Fig. 1a), oral laboratory presentation (Fig. 1b), and overall quality of student dissection (Fig. 1c). Therefore, the type and timing of diagnostic imaging implementation had no influence on the students’ academic achievement for these course components.

### Qualitative results

Through qualitative coding and thematic triangulation, a variety of themes and sub-themes were explored. As demonstrated in Table 3, one of the main themes arising from the focus group sessions was *Factors Influencing the Dissection Experience*. This was further broken down into the following sub- or analytical-themes which best describe the students’ perceptions of their experience with integrating diagnostic imaging into anatomical dissection; the overall impact of diagnostic imaging on the dissection experience, differences of the PSDI experience, and suggestions for curricular change. Following the qualitative analysis, it was evident that the students provided with pre-mortem DSDI felt that they were able to plan an approach to their dissection (Table 3, quotes 1–2) and correlate the pathologies observed in the diagnostic imaging provided with their findings during the dissection process (Table 3 quotes, 3–4). Furthermore, students expressed that they felt that they were able to improve their radiological reading through this experience (Table 3, quote 5) while simulating a surgical experience (Table 3, quotes 6–7). Though students in the PSDI group found that they were generally able to correlate pathologies seen in their imaging with their dissection, despite the imaging not being donor-specific (Table 3, quote 8), they also discussed a hindered experience given the challenges associated with linking the PSDI imaging findings directly to their dissection (Table 3, quote 9–10). In addition to this, there was also discordance in how students felt the integration of their type of imaging impacted their overall experience. Students in the PSDI groups related the AFS dissection experience to their experience as a first-year medical student, whereby they attended the anatomy laboratory and conducted dissections not knowing what they would find (Table 3, quote 11).

**Table 3:** Factors Influencing the Dissection Experience

**Impact of Diagnostic Imaging on Dissection Experience**	
**Imaging Aids in Dissection Planning**	1. “It’s nice to have an idea of what’s going to be happening, or what you are going to be seeing.” *–DSDI-DG Student*
	2. “On our imaging... [the donor’s] right kidney looked a little infarcted, so when we went there we weren’t expecting a big thing and low and behold it was a tiny thing.” –*DSDI-DG Student*
**Ability to View Pathologies and Correlate with Dissection**	3. “Yeah. For us, it just like affirmed what we were seeing in the anatomy. So with the lung, it was very emphysemic, so when we were taking it out, it was attached to the pleura. It sort of made sense. And then when we were doing the laminectomy, there was also a lot going on there. So I think it was nice to know that we do have pathology and seeing the radiographic images beforehand just confirmed that. So yeah.” –*DSDI-DG Student*
	4. “We only got the generic ones, so it’s not exactly the same. But I think if we had seen them from the beginning, like we said, we would have expected the adrenal, and we would have expected some mass in the lung. We would have expected some things, and then it would be nice to correlate this as we were finding these things” *–PSDI-DG Student*
**Integration of Imaging Aids in Radiological Reading Skills**	5. “I’d say [the images] really helped my radiological readings quite a bit. There were lots of things where I would look at the images and I had an idea of maybe what this finding is, and in fact, when you go dissect it, you realize oh, actually, no...” *–DSDI-DG Student*
**Integration of Imaging Simulates Surgical Experience**	6. “… it’s a bit like an actual operating clinical kind of scenario … when you don’t have the imaging, it’s kind of just like you’re going in blind, and everything surprises you. So, if you have [the imaging at the beginning], it kind of helps put things in perspective …” –*PSDI-DG Student*
	7. “I think it’s just having the whole thing like it creates a story, it helps create the view.” –*DSDI-DG Student*
**Differences of the Pathology-Specific Diagnostic Imaging Dissection Experience**	
**Positives and Challenges Linking Pathology Specific Diagnostic Images with Dissection**	8. “There was also learning around [the] image [for example it] tells us that it’s, you know, like a parenchymal versus, like, an air space lesion. And then these are the kinds of things you should look for. What do you see in the mediastinum? What does that tell you? ... So that was super informative for me. *–PSDI-DG Student*
	9. “I had a tough time making the relation between the images and what we were doing...” *–PSDI-DG Student*
	10. “Something that if we had donor-specific imaging, then all these findings that we know we’re not sure whether they’re pathologic or not, or just sort of a variation, I think then it would have been nice to have that imaging to actually just say oh, let’s just look. What is that we found on the imaging? Is it something that looks so different to us?” *–PSDI-DG Student*
**Comparable to First Year Medicine Experience**	11. “Whereas going to this felt like Med 1. It felt like a Med 1 experience, which is just going at the cadavers you don’t know the history and we just dissect.” –*PSDI-DG Student*
**Curricular Suggestions**	
**Format Integration is Optimal for Senior Medical Students**	12. “I think it’s nice to do it after we’ve gone through our surgical clerkships because you’ve seen things, and then now you understand it. Before, I feel like even in my, like, junior clerkship and my core, I didn’t really grasp the concept of planes and things like that. I felt like that was more consolidated, so when you come now, you have those concepts.” *–DSDI-DG Student*
**Earlier Integration is Possible/Beneficial**	13. “I mean, if …even in Med 1 or Med 2, I would have liked … having pictures related to my own body and ... just having a resident sit with five people or four people who are dissecting the body, like what we did right now would help a lot, just to remember one or two things from that specific body.” *–DSDI-DG Student*
**Appreciation for Small Group Format to Assess Diagnostic Imaging**	14. “No one ever takes the time to sit down with you. They just like…in the hospital, they scroll through it all ... and you’re like, what am I looking at? So [the radiologist interaction] was super useful.” *–DSDI-DG Student*
	15. “I thought [the question and answer session] was actually kind of interesting and sort of informative to actually how [sic] read more scans than actually our own scan that we had, which was kind of cool because we don’t really get…like, the teaching that we get isn’t kind of that focused, and it gave us a lot of really cool tricks and techniques.” –*PSDI-DG Student*

*DSDI-DG* Donor specific diagnostic imaging dissection group, *PSDI-DG* Pathology specific diagnostic imaging dissection group, *Med 1* Year one of medicine, *Med 2* Year two of medicine

The focus group sessions also provided evidence to suggest that students in both DSDI and PSDI groups appreciated the structure of diagnostic imaging integration with anatomical dissection and would like to see its implementation formally into the medical curriculum. The format of pre-mortem DSDI implementation into the AFS course was understood to be optimal for senior medical students given their clerkship experiences (Table 3, quote 12); however, students also believed that early integration into the first- and second-year anatomical dissections would be plausible and more engaging with appropriate curricular tailoring (Table 3, quote 13). During the students’ first year of medical school at McGill University, radiology is integrated into the dissection-based anatomy laboratories by way of a 10–15 min tutorial presentation given by a radiologist on the normal anatomy and common pathologies associated with the anatomical system being studied in that unit.

Lastly, both DSDI and PSDI groups agreed that the most optimal way to teach radiographic interpretation would be to receive imaging prior to the start of dissection and continue to use it throughout the dissection while learning in a small group format similar to the question and answer period with radiology residents that was provided as part of the AFS course (Table 3, quotes 14–15). This would allow for the comparison of anatomical structures and pathological findings from 2D imaging to their 3D anatomical presentations in the laboratory while promoting a more collaborative approach to the teaching of radiology.

As demonstrated in Table 4, the second main theme arising from the focus group sessions was the *Influence of Diagnostic Imaging on Professional Mindset*. This was further broken down into the following sub- or analytical-themes; the notion of emotional attachment to the body donor and becoming more “patient-minded” through the use of pre-mortem DSDI, the notion of emotional detachment from the body donor and being more “body-minded” as a result of the PSDI experience, and the impact that the diagnostic imaging had on all participants’ medical training for their future careers. Students with access to pre-mortem DSDI expressed an emotional attachment to their body donors for multiple reasons. The combination of the case vignette along with the early access to the pre-mortem DSDI brought the patient to life. The case vignette provided a patient storyline and the information given on the pre-mortem DSDI allowed the students to plan their dissection just as they would use the patient history and diagnostic imaging to plan for surgery. For these reasons, this more closely simulated the role of a physician, and as such, encouraged students to adopt a “patient-minded” approach to their anatomy dissection (Table 4, quote 1). In addition, students in the DSDI dissection group often referred to their body donor as “*our patient*” rather than their cadaver in sub-conscious and conscious ways (Table 4, quotes 2–4). On the contrary, the students provided with PSDI discussed an experience that led them to feel emotionally distant from their body donors and as a result, adopted a more “body-minded” approach. This was in part due to the fact that students in the PSDI groups felt that they (1) lacked clinical information on their body donor by being provided with PSDI (Table 4, quote 5) and (2) felt that their AFS course experience was more of an exploratory dissection of the body donor since their imaging was not specific to their body donor. The PSDI dissection group brought up the notion that exploratory dissection altered the architecture of the body (Table 4, quote 6). These discussions therefore provided evidence to suggest that the integration of pre-mortem DSDI may allow students to assume a professional mindset in the anatomy laboratory and foster additional characteristics important for physicians such as empathy and respect.

**Table 4:** Influence of Diagnostic Imaging on Professional Mindset

**Relating to the Body Donor – “Patient -Minded”**	
**Imaging Integration Provides Patient Storyline**	1. “I think just to add to that, I think in addition to the imaging, like having the background, the history, as I mentioned earlier adding more and more detail of past medical history brings this person to life. Like, having seen the patient, you see what they go through, what they come to, and that you see this patient in front of you, knowing what they had gone through and what brought them here.” *–DSDI-DG Student*
**Addressing the Body Donor as “Patient”**	2. “I actually noticed that I now … call our donor a patient, and I think in Med 1 and Med 2, I didn’t do that … I say ‘our patient’ now, and I think that might be because of imaging.” *–DSDI-DG Student*
	3. “For me, I felt when I was approaching the patient, I found that I was trying to be very safe about structures, just like you would be in an OR, where you’re trying to identify, you want to make sure you don’t cut it … versus if it was just a cadaver, I’m looking for a nerve and I couldn’t care less.” *–DSDI-DG Student*
	4. “I think all those things contribute to the fact that you now view the patient as more…or the donor as more of a patient. I would have also liked to seen more past medical history of the patient because that’s something that you can provide to the students without needing any imaging. It will also help us to, like, approach a patient in a different way. If we had known that the patient had a total knee replacement, just based on past medical history, then we would be more cognizant of the scar tissue around his knee, for example.” *–DSDI-DG Student*
**Detachment from the Body Donor – “Body-Minded”**	
**Lack of Clinical Information**	5. “Another thing, at table seven, not only were there generic images, but it felt like generic issues too because we didn’t see any of that on the cadaver. We couldn’t correlate any of that.” *–PSDI-DG Student*
**Process of “Exploratory Dissection”**	6. “I feel that just the process of the dissection has completely changed the entire architecture of the body to the point that, like, the weight is completely different. Like, she weighs less, we have a hard time to turn her, you know. Everything is destabilized, and so definitely, it feels very different at this point…” *–PSDI-DG Student*
**Simulation of Future Practice**	
**Simulating the OR Experience**	7. “It was touched on earlier. When we were given clinical vignettes or given imaging, it’s very much like going into the OR. We understand the patient’s story. We get to flip through the imaging before the OR, and that’s what it feels like.” *–PSDI-DG Student*
	8. “Yeah. I think the vignette definitely helps because it’s what you do in the hospital. If you’re in emergency or whatever, and you get kind of the story, the patient age or what their medical problems are and what the presenting issue is, and already you start to formulate your impression of what this person looks like, what they’re coming in with, what’s going on. So I think it for sure helps to humanize it.” *–DSDI-DG Student*
**Envisioning Oneself as a Surgeon/Physician**	9. “… makes us all want to be in more the role as an anatomical pathologist, where first of all that’s the physician role. Second, you’re going through with your whole team trying to figure out what every pathology could possibly be linked to in terms of the cause of death and having all of the imaging also sort of puts you more into that way of thinking.” *–DSDI-DG Student*

*DSDI-DG* Donor specific diagnostic imaging dissection group, *PSDI-DG* Pathology specific diagnostic imaging dissection group, *Med 1* Year one of medicine, *Med 2* Year two of medicine, *OR* Operating room

Lastly, when discussing the impact of integrating diagnostic imaging with anatomical dissection on their medical training, both student groups expressed that this experience, regardless of the type of imaging provided, positively impacted their preparation for future practice. In either scenario, students in the DSDI and PSDI dissection groups felt that having access to diagnostic imaging and a case vignette, which correlated to structures they could anticipate seeing in their dissection, more closely simulated the operating room experience and the various steps involved in preparing for surgery (Table 4, quotes 7–8). Additionally, students felt that the information provided allowed them to assume the role of an “anatomical pathologist” or envision themselves as a surgeon/physician throughout the dissection process, possibly contributing to the students’ professional identity (Table 4, quote 9).

## Discussion

### Benefits of utilizing pre-mortem DSDI in anatomical education

The novelty of working with pre-mortem DSDI demonstrated profound impacts on the student-donor relationship, encouraging a more empathic, respectful and patient-like approach to dissection. There was further recognition, that alongside the development of these soft-skills, students recognized the benefits of this imaging intervention on their ability to relate diagnostic imaging to the gross anatomy in front of them, all of which provided application for their future clinical practices, and provoked added interest to incorporate pre-mortem DSDI into the junior medical education curricula. This study is the first of its kind to implement pre-mortem imaging of body donors into a dissection-based medical anatomy course. Studies similar in nature have evaluated the integration of donor-specific imaging in the anatomical study of body donors; however, this imaging has previously only consisted of post-mortem scans [7, 10, 11, 13, 14]. The quality of post-mortem imaging presents various challenges with the production of imaging conducted either pre- or post-embalming [7, 13–16]. The process of embalming produces scanning artifacts that deteriorate image quality, causing the interpretation of post-mortem imaging to be challenging [15]. Chew and colleagues [7] described common difficulties, including airspace filling and pleural effusions in the chest, free peritoneal fluid in the abdomen, and blood clotting in the blood vessels [7]. In the current study, since both PSDI and DSDI dissection groups received diagnostic pre-mortem imaging (PSDI not being donor specific), this eliminated all major issues with imaging quality. Overall, student survey responses indicated that regardless of their assigned diagnostic imaging-type, they all agreed that the quality of imaging received was optimal for viewing and easy to interpret. This finding was also consistent in focus group sessions whereby students had no complaints or negative feedback concerning the quality of the imaging received. Other challenges associated with post-mortem imaging include the time needed to acquire the imaging and the cost associated [15]. Pre-mortem imaging however presents as a highly advantageous educational resource as it eliminates many of the expressed concerns relating to post-mortem imaging. Donor imaging acquisition for the purposes of this study was free-of-charge and organized in collaboration with the Department of Diagnostic Radiology at McGill University without difficulty. These factors are important to consider when implementing a similar intervention into an anatomical dissection-based course. An additional benefit to pre-mortem body donor imaging is the access to a variety of modalities such as CT scans with contrast agents, magnetic resonance imaging (MRI), X-ray, positron emission tomography (PET) and angiograms. Unfortunately, imaging quality is compromised with various imaging-types for post-mortem scanning of body donors, specifically of the abdomen where the majority of pathology is seen [15]. Post-mortem imaging is particularly limited to CT and MRI scanning since these methods are best suited for embalmed body donors [15]. Arguments that post-mortem imaging helps with the fidelity of the location of the organs during embalming up until the process of dissection, can only be justified for the intra-peritoneal organs. All solid and fixed organs (i.e. intra-peritoneal organs) keep their position from pre-mortem to post-mortem examination. For these reasons, implementing pre-mortem imaging into the anatomical study of body donors is beneficial to medical students providing increased exposure to multiple imaging modalities in the anatomy laboratory.

### Advantages of donor-specific over pathology-specific diagnostic imaging

As radiological imaging continues to be incorporated into anatomical medical curricula, there is a need to evaluate the effectiveness of this tool on anatomical understanding. Fortunately, Lufler and colleagues [10] have used validated testing to assess the impact of the use of cadaveric post-mortem CT scans on student performance in a gross anatomy course. In their investigation, students who chose to use the CT images scored significantly better on all assessments, which included radiological imaging interpretation, in comparison to their counterparts whom did not use the CT imaging to study. Furthermore, within the group of students that used the CT imaging, a subset of these students received imaging that was specific to their assigned body donor. Upon analysis, students who used the donor-specific CT images did equally as well on assessment scores as those students provided with imaging that was generic [10]. This finding is consistent with our results whereby student academic assessment scores across the oral presentation, dissection quality, and oral examination were not influenced by whether or not students received pre-mortem DSDI. Despite there being no differences concerning academic achievement in our investigation, statistically significant differences were found in the survey items assessing the complementarity and relevancy of imaging with dissection. The later result was particularly interesting to the authors as other investigators have observed no differences between students exposed to either post-mortem, donor-specific scans or generic CT imaging [10]. With this in mind, the findings of the current investigation lead the authors to believe that the specific use of pre-mortem DSDI had profound advantages over students receiving post-mortem DSDI scans as seen in the investigation by Luftler and colleagues [10]. Furthermore, student feedback regarding their dissections was greatly enhanced given the pre-mortem DSDI. It is evident therefore that although DSDI and PSDI students performed equally on related course assessments, students with access to pre-mortem DSDI gained more practical knowledge relating to the surgical process, body donor pathology correlation with imaging and dissection planning than their PSDI counterparts. The authenticity of these pre-mortem images particularly afforded the students in the DSDI groups the opportunity to plan their anatomical dissection according to the donor-specific anomalies seen just as they would when planning a surgical intervention.

### Pre-mortem DSDI affords the development of both hard and soft skills

Aside from increasing the students’ knowledge of anatomy, many dissection programs find the balance between instilling empathy and navigating clinical detachment in the anatomy laboratory challenging. In many cases, medical students have reported reduced empathy towards their body donor over the course of dissection [28]. Through the incorporation of pre-mortem DSDI alongside a case vignette, students were able to dissociate this dissection experience as a simple act of learning and alter their perceptions of the body donor from an educational resource to that of a “patient.” This finding is supported by another investigation which showed that students who utilized DSDI in their anatomy learning benefited by exhibiting more empathic, respectful, and professional behaviours towards the body donors [13]. Although PSDI dissection group students received imaging and a case-vignette related to their donor, the fact that the imaging was not donor-specific created a dissociation whereby they did not express the same consideration of their body donor as did students in the DSDI dissection group. Particularly, students in the DSDI dissection group not only expanded their experience beyond acknowledging their body donor as a patient, but rather thought of their body donor holistically, taking into account the history of what their body donor once was. In fact, throughout the entirety of the focus group sessions conducted, there was a notable difference in the choice of wording and view of the body donor between DSDI and PSDI dissection groups. DSDI dissection groups used words like *“bringing the person to life”* and consistently used the term *“patient”* to refer to their body donors. The use of pre-mortem DSDI evoked life-like reflections made by students which were facilitated by the authenticity of these images being pre-mortem. Furthermore, the resonance of empathy and the patient storyline created by incorporating pre-mortem DSDI into anatomical dissection imparted pertinent values which are advantageous to the training of physicians [29]. Students in the PSDI dissection group instead frequently used the terms *“body”* and *“cadaver”* to refer to their body donor. This aspect of mindful reflective practice in relation to pre-mortem DSDI integration has yet to be conveyed by previously published works.

### Shifting medical student perspectives to become more patient-centered

The concept that a cadaver is “dual in nature” as both a person and a specimen has been explored in the work by Goss and colleagues [30]. Supported by previous work of Hafferty [31], Goss and colleagues [30] described that students will adopt a view of their cadaver during dissection that “aligns with their emotional needs and professional goals.” According to their findings, there existed a spectrum; those who viewed the donor as a person throughout the course were labelled as “person-minded” and those students whom rarely acknowledged the donor as a person were described as being “specimen-minded.” The centre of the spectrum is where the majority of students fell whom consistently recognized going back and forth between both schools of thought. Students in the “person-minded” group were said to adopt this view of creating an environment to explore emotion and empathy and to develop respect for the body. In contrast, students in the “specimen-minded” group adopted this view in order to detach themselves from the emotional and moral concerns and focus on the technical side of dissection [30]. The findings of the present study support the notion that “person-mindedness” can be facilitated by incorporating tools such as pre-mortem DSDI into anatomical dissection. Students in the DSDI dissection group similarly embraced a humanistic view of their body donor putting respect, reflection, and emotion at the forefront of their dissection experience. This type of reflective practice is a crucial component to becoming a medical professional as resilience is acquired through the development of professionalism and self-awareness [32, 33]. Additionally, by incorporating reflection into anatomy curricula, students are made aware that these concepts are equally as important as their anatomical education in their professional development towards becoming a physician [34]. Overall, the use of pre-mortem DSDI in this study facilitated an experience that mimicked a clinical setting making the donor feel more like a “patient” and making the anatomy laboratory environment a space for reflection.

### Potential for earlier integration of pre-mortem DSDI into medical curricula

The timing of the implementation of radiology and anatomy into medical curricula is an important topic worth investigating. One of the difficulties with integrating radiology and anatomy in the early periods of a medical curriculum is overcoming the students’ low confidence and experience when it comes to radiological interpretation [13]. Students in their senior years of medical school have more experience, particularly following their clerkship rotations, and are now ready to appreciate and engage in the incorporation of radiology with anatomy. In the present investigation, while both DSDI and PSDI dissection group students agreed that fourth-year medicine was an appropriate time to integrate these fields, students in the DSDI group expressed they would have liked to see integration earlier in their medical curriculum given the impactful experiences they were engaged in during the AFS course. This finding is consistent with various other studies that have displayed that students enjoy having radiology incorporated earlier into their medical curricula [8, 9]. By implementing radiology into pre-clinical years of the medical curriculum, students will have the opportunity to learn more about the specialty of radiology, better appreciate the relationship between radiology and anatomy, increase their performance on radiology-specific assessments [8, 9], and are more likely to choose a radiology elective course in later years of medical school [8, 35, 36]. Students mentioned during the focus group sessions that the format used in the present investigation could be an alternative to the current format implemented in the earlier years of their medical curriculum. In the first-year format, anatomy is typically seen during dissection in the anatomy laboratory, and the presentation of anatomical structures using radiology is presented by radiologists at the end of a given dissection. It is important to note, however, that all students in this investigation thoroughly enjoyed the small group format utilized in the AFS elective course whereby discussions were led by the guidance of a radiology resident amidst the dissection time. Overall, the potential of this investigation to be integrated into first and second years of the medical curriculum is of interest to senior students reflecting on their junior years in medical school and could allow students the opportunity to integrate their anatomy and radiology education earlier in medical school.

### Future directions

Lastly, it is of importance to recognize the ability of pre-mortem DSDI to revolutionize the way in which anatomy is taught. Through the acquisition of the body donors’ consent to retroactively access pre-mortem imaging across various life-periods, students gain the ability to observe and learn to interpret images of diagnostic-quality and directly relate the 2D anatomy in these images with the 3D anatomy presented by their respective body donor in the anatomy laboratory. Other possibilities expand to the ability to track pathology changes over the course of one’s life. Pre-mortem DSDI provides the capacity to track the structural and functional manifestations of the disease that a particular individual may have endured or provide advanced discussions surrounding surgical approaches through the procurement of pre- and post-operative body donor scans. Each of these scenarios cannot be as easily replicated using other imaging interventions or display the same quality that pre-mortem DSDI affords. It is of the authors’ beliefs, therefore, that the current investigation demonstrates the ability of pre-mortem DSDI to facilitate the development of both hard and soft skills associated with physicianship.

## Limitations

Though the current study provides evidence for a novel approach to integrating pre-mortem imaging into anatomical dissection within a medical course, there are a few limitations to consider. Firstly, all data was collected from a single institution and from one iteration of the AFS course. Though 74% of the AFS students (*n* = 26) consented to participate in this investigation, the authors recognize that due to the smaller class size (*n* = 35), the comparative group sizes were also small, and not equal in numbers. The small sample size can be attributed to the fact that AFS is an elective course with a capped class size. Due to the nature of the course being self-selected, this could also be a contributing factor influencing how students may perform in the course. Of note, none of the academic assessments in this investigation included imaging-related questions and all questioning was related to the gross anatomy of the body donor. This was selected since the AFS course learning objectives are set to develop various facets of anatomical knowledge, not solely imaging interpretation, in preparation for refining students’ knowledge and skills for future practice. Interventions that have incorporated anatomy and radiology together in medical curricula have seen greater achievement on academic assessments that included imaging interpretation, namely using cross-sectional CT images [37] and identification of anatomical structures on various types of radiological imaging, both short and long-term [38]. In line with what was seen in this investigation, other investigations have found that, other than increasing academic scores, the integration facilitates greater clinical relevancy [39], and enhances student knowledge to become a better physician [40, 41]. Lastly, potential confounding bias may be present with the introduction of two variables in this study; the incorporation and comparison of different diagnostic imaging types (DSDI and PSDI) and differences in the timing of the receipt of the various diagnostic imaging types. It is therefore difficult to fully determine whether the students’ experiences were a result of the timing alone, the type of diagnostic imaging alone, or a combination of the two. The PSDI imaging was provided to students half-way through the course prior to beginning their abdomen dissections. Given that the majority of donor pathologies in the PSDI-collection were abdomen-related, this seemed to be an optimal time to introduce the PSDI without disadvantaging this group of students academically. This further avoided any early pre-planning of their approach to the dissection on pathologies seen in the PSDI, which were only representative, yet this still afforded students the opportunity and the experience of relating their imaging with the relevant donor pathologies prior to and throughout the dissection. With this in mind, it is the belief of the authors that the observed results are more likely a product of the type of diagnostic imaging intervention (i.e. DSDI versus PSDI), as opposed to the timing of the receipt of diagnostic imaging.

## Conclusion

The innovative nature of pre-mortem imaging incorporated into the anatomy laboratory provides students with an authentic experience, true to their future professional career. To our knowledge, this investigation is the first of its kind to demonstrate the advantages of implementing pre-mortem DSDI into a dissection-based medical anatomy course. Successful integration of diagnostic imaging was attributed to the imaging being donor-specific, the accompaniment of a patient case vignette, and early receipt of the imaging in the course scheduling which allowed for continuous correlation between findings on the diagnostic imaging and the students’ dissection. The DSDI dissection groups expressed a more humanistic approach to the body donor, often replacing the term cadaver with “patient,” and demonstrated empathy even during the dissection process. The occurrence of these phenomena demonstrate that the implementation of pre-mortem DSDI helped to create a simulated clinical experience for the students. It is therefore evident that combining pre-mortem DSDI into anatomical dissection has positive effects on the training of medical students in relation to their knowledge and their development of personal and professional characteristics relating to clinical practice. Future studies on this topic should aim to explore the extent to which students can develop empathy and professionalism towards the body donors, contributing to their professional identity and further investigate the effectiveness of earlier implementation of diagnostic imaging with anatomical dissection within a medical curriculum.

## Data Availability

The datasets generated during and/or analysed during the current study are not publicly available to protect student anonymity as study participants, particularly the students’ assessment scores and the students’ comments/discussions transcripts for qualitative coding. Datasets can be made available from the corresponding author on reasonable request.

## Abbreviations

AFS: Anatomy for surgeons
CT: Computed tomography
DSDI: Donor-specific diagnostic imaging
DSQ: Dossier Santé Québec
FMD: Fundamentals of Medicine and Dentistry
M: Mean
Med 1: Medicine year 1
Med 2: Medicine year 2
Md: Median
MRI: Magnetic resonance imaging
OR: Operating room
PET: Positron emission tomography
SD: Standard deviation
